# A highly sensitive vertical plug-in source drain high Schottky barrier bilateral gate controlled bidirectional tunnel field effect transistor

**DOI:** 10.1371/journal.pone.0285320

**Published:** 2023-05-19

**Authors:** Xi Liu, Mengmeng Li, Meile Wu, Shouqiang Zhang, Xiaoshi Jin

**Affiliations:** School of Information Science and Engineering, Shenyang University of Technology, Shenyang, China; Federal University of ABC, BRAZIL

## Abstract

In this article, we propose a highly sensitive vertically plug-in source drain contacts high Schottky barrier based bilateral gate and assistant gate controlled bidirectional tunnel field Effect transistor (VPISDC-HSB-BTFET). It can achieve much more sensitive forward current driving ability than the previously proposed High Schottky barrier source/drain contacts based bilateral gate and assistant Gate controlled bidirectional tunnel field Effect transistor (HSB-BTFET). Silicon body of the proposed VPISDC-HSB-BTFET is etched into a U-shaped structure. By etching both sides of the silicon body to form vertically plug-in source drain contacts, the source and drain electrodes are plugged into a certain height of the vertical parts of both sides of the U-shaped silicon body. Thereafter, the efficient area of the band-to-band tunneling generation region near the source drain contacts is significantly increased, so as to achieve sensitive ON-state current driving ability. Comparing to the mainstream FinFET technology, lower subthreshold swing, lower static power consumption and Higher I_**on**_−I_**off**_ ratio can be achieved.

## Introduction

The research on the basic unit of integrated circuits is based on two aspects. One is to improve the integration density, and the other is to improve the device performance. It is important to reduce the size of the basic unit of the integrated circuit as much as possible. Multi-gate MOSFET is impressive in sub-30nm technology nodes [1, 2]. However, it is necessary to use expensive millisecond annealing technology to achieve abrupt junctions at the nanometer scale [3]. Schottky barrier MOSFET (SB-MOSFET) forms shallow Schottky barrier instead of the p-n junction barrier of MOSFET [4–6]. The metallic source/drain (S/D) architecture holds the advantage to relax severe constraints imposed to conventional implanted S/D [7]. For p-type SB MOSFETs, the height of Schottky barrier for holes in valence band φ_Bp_ is set to be much smaller than the one for electrons in conduction band φ_Bn_. For a shallow Schottky barrier height, the thermionic emission current is always smaller than in the ideal 0 eV barrier height case, thereafter, the subthreshold swing (SS) of SB MOSFETs is larger than 60 mV/decade, the inability of subthermal SS through a Schottky barrier without considering others physical mechanisms such as band-to-band tunneling (BTBT) has been proved by a simple potential mapping method, thereafter Although SB-MOSFET is easier to be manufactured than conventional MOSFETs in nanoscale process, these physical mechanisms also leads to performance degradation such as the lower ON-OFF current ratio and the forward-reverse current ratio [8, 9]. For performance improvement, several novel devices are purposefully developed, among which TFET (tunnel field effect transistor) is the most representative, it utilizes BTBT as the current conduction mechanism which can realize more sensitive subthermal SS [10–14]. Unfortunately, to realize smaller SS, abrupt junction in TFET also has to be formed which is similarly to MOSFETs [15–18]. Besides, the current driving ability is much poor than MOSFETs, in the reversely biased state, BTBT induced leakage current will be significantly increased, even larger than the forward current. To avoid doping process in TFET, doping-less tunnel FET or charge plasma based nanowire TFET are proposed [19–23]. For bidirectional operation, a bidirectional tunneling field effect transistor (BTFET) based on high Schottky barrier (HSB) such as HSB-BTFET and A novel high-low-high Schottky barrier based bidirectional tunnel field effect transistor (HLHSB-BTFET) [24, 25]. However, due to that band-to-band tunneling is the main current generation mechanism of HSB-BTFET or HLHSB-BTFET, similar as other type of TFETs, high source-drain impedance is formed, and the forward ON-state current driving ability is seriously limited. In order to significantly increase the forward conduction current without leads to integration degradation, we proposed a highly sensitive vertically plug-in source drain contacts high Schottky barrier Based Bilateral Gate and Assistant Gate Controlled Bidirectional Tunnel Field Effect Transistor (VPISDC-HSB-BTFET). The efficient source drain contacts area is significantly increased without increasing any extra chip area, the source and drain contacts are plugged deeply into the silicon body, which maximizes the contact area. Maximizing the contact area increases the number of electron-hole pairs generated at the same voltage by band-to-band tunneling phenomena, and much higher ON-state current can be generated. Compared to the previously proposed HSB-TFET, the VPISDC-HSB-BTFET can realize higher integration, lower subthreshold swing, much smaller reverse bias leakage current, higher on state current and I_on_-I_off_ ratio.

### Design conception and operating principle

**Fig 1:**
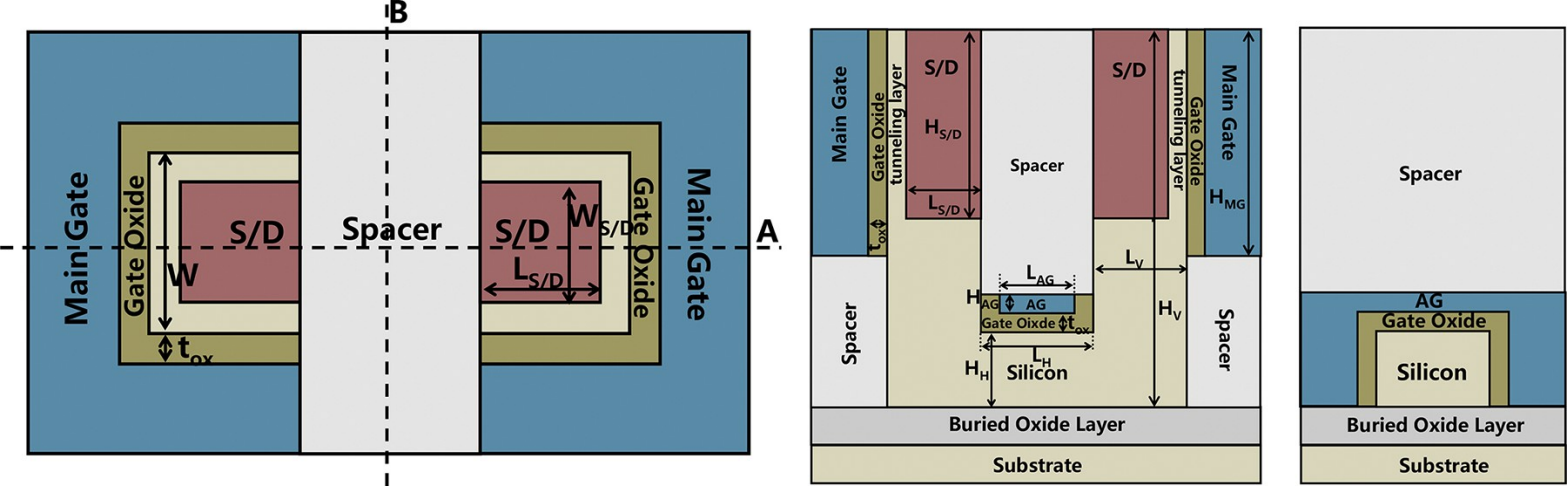
(a) the top view of the proposed VPISDC-HSB-BTFET, (b) the cross views along cut line A in (a), (c) the cross views along cut line B in (a).

Fig 1(A) is the top view of the proposed VPISDC-HSB-BTFET, Fig 1(B) and 1(C) are the cross views along cut line A and B in Fig 1(A), respectively. As shown in Fig 1(A) and 1(B), the device structure is symmetric, and the source / drain regions are interchangeable. As shown in Fig 1(A), the main control gate is a pair of brackets on both sides of the source and drain to control the silicon near the source and drain from three directions. Enhance the control of source and drain. Fig 1(B) shows that the silicon is etched into a U-shaped structure. By etching both sides of the silicon body again, the source and drain electrodes are plugged into a certain height of the vertical parts of both sides of the U-shaped silicon. Fig 1(C) shows the assistant gate presents inverted U-shaped structure, which is similar to the gate structure of FinFET and controls the three sides of the horizontal part of the bottom of the U-shaped silicon body. L_V_ and L_H_ are the lengths of the vertical and horizontal portions of the U-shaped silicon along source-to-drain direction, respectively. W is the width of the horizontal part of the U-shaped silicon. L_S/D_ and W_S/D_ are the lengths and widths of source/drain contacts, respectively. H_S/D_ is the height of the source / drain contact. H_H_ and H_V_ are both of the thickness of the horizontal and vertical portions of the U-shaped silicon. t_ox_ is the thickness of gate oxide, H_AG_ is the height of the assistant gate. L_AG_ is the width of the assistant gate. H_MG_ is the height of the main gate.

Considering that the subthermal subthreshold swing can be obtained by band to band tunneling on a sharp metallic junction, metallic junctions based high Schottky barrier are formed in both source and drain regions. In general, SB-MOSFET generates thermionic emission current through a relatively low Schottky barrier as the physical mechanism of forward current supply. The VPISDC-HSB-BTFET proposed in this paper forms a high Schottky barrier near the center of the band gap. This can block the Schottky barrier thermionic emission current to a large extent. Instead, it increases the generation of band-band tunneling current as the turn-on mechanism of the device. Considering that the total amount of the tunneling current is related to the total volume of the silicon region where the tunneling effect can occur and the magnitude of the electric field in the tunneling region, the tunneling region should be designed as large as possible. As shown in Fig 1(B), the channel part is designed with a recess structure, which is a U-shaped channel. Plug-in source drain contacts are designed for VPISDC-HSB-BTFET for ON-state current enhancement by enlarge the efficient area of the band-to-band tunneling region to achieving the maximization of carrier generation and ensuring that the source-drain distance is relatively large, which effectively preventing reverse leakage. By increasing the height of the vertical portion of the U-shaped silicon region, the total volume of the tunnel layer can be greatly increased without increasing the total chip area occupied by the device. The inverted U-shaped assistant gate controls the flow of carriers in the central channel of the device. The horizontal portion of the U-shaped silicon region allows electrons to pass through and blocks holes.

### Analysis and discussions

The characteristics of the proposed VPISDC-HSB-BTFET have been verified by device simulation using SILVACO Tools [26]. Physical models such as quantum confinement model, Shockley-Read-Hall recombination model, Auger recombination model, mobility model, band gap narrowing model, a standard band to band tunneling model, Fowler-Nordheim tunneling model are all turn on [27–29].

**Fig 2:**
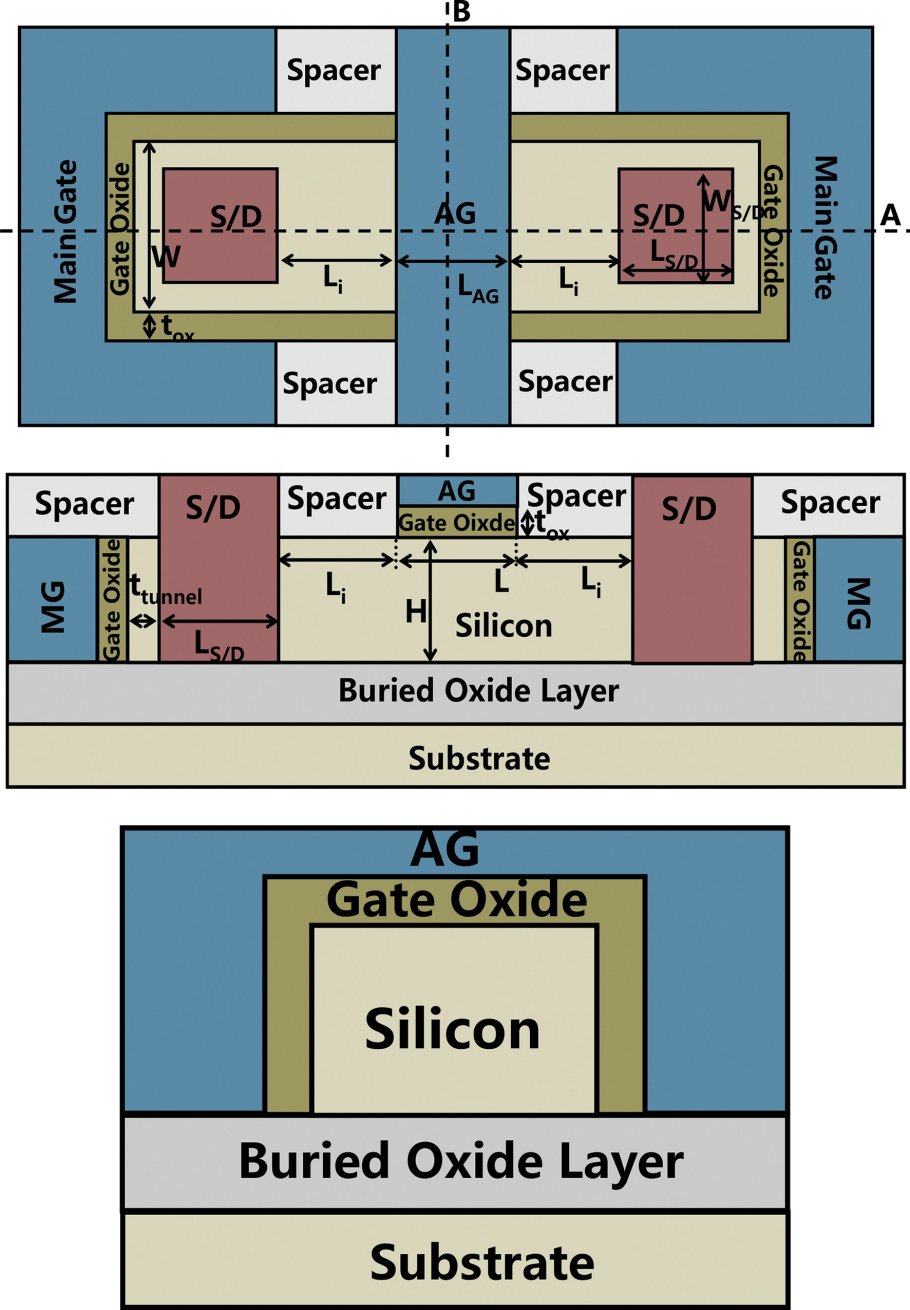
(a) the top view of the proposed VPISDC-HSB-BTFET, (b) the cross views along cut line A in (a), (c) the cross views along cut line B in (a).

In order to verify the performance of the device, we compare the newly proposed VPISDC-HSB-BTFET and HSB-BTFET. Fig 2(A) shows a schematic view of the top view of HSB-BTFET, Fig 2(B) and 2(C) are the cross view of HSB-BTFET along the cut line A and B in Fig 2(A). The I_DS_-V_GS_ characteristics of HSB-BTFET and VPISDC-HSB-BTFET are compared in the same simulation environment.

**Fig 3:**
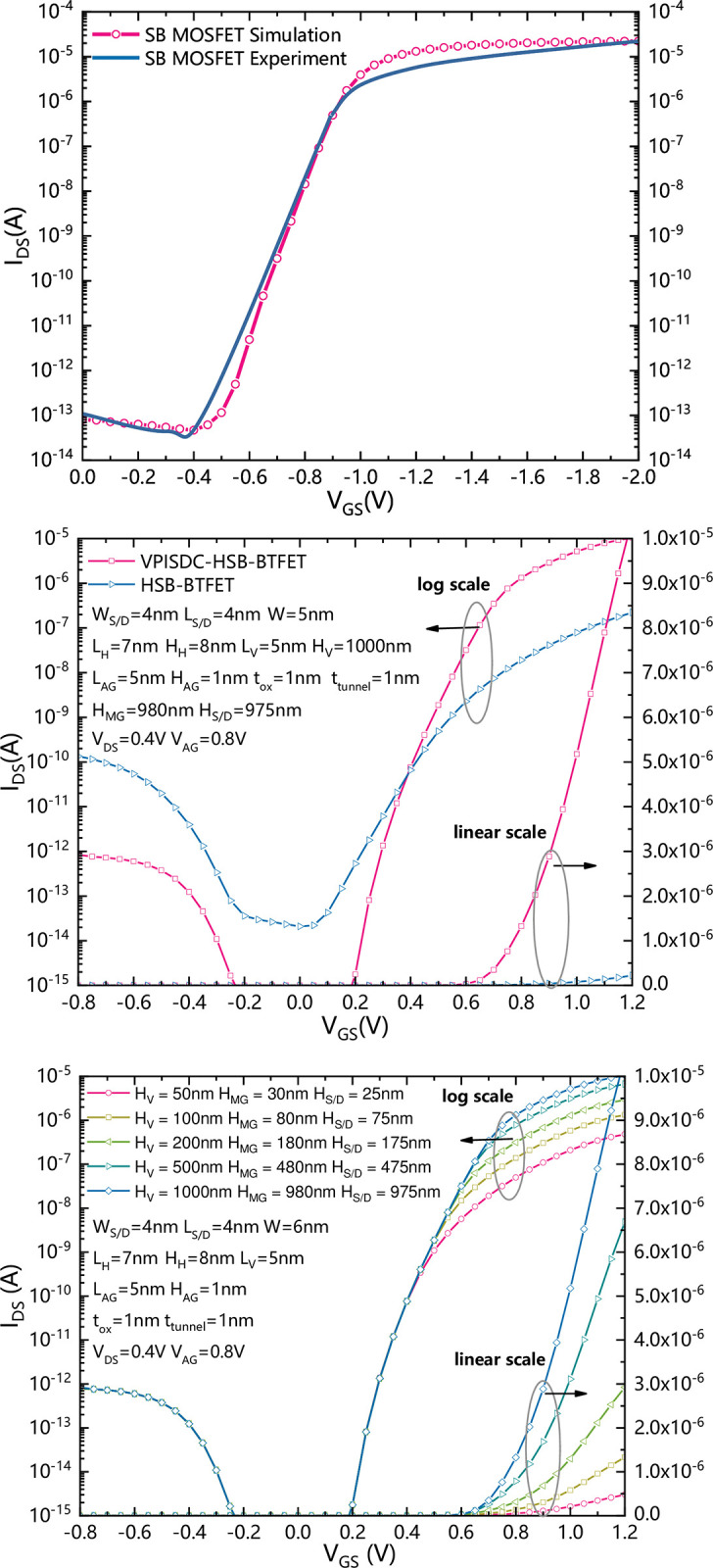
(a) Calibration between a simulated SB-MOSFET and an experimental SB-MOSFET. (b) Transfer characteristics comparison between HSB-BTFET and VPISDC-HSB-BTFET. (b) The I_DS_-V_GS_ characteristic curve of different vertical channel heights.

Fig 3(A) shows a calibration of simulation. To ensure the accuracy of the simulation, we calibrated the transfer characteristics of a simulated SB-MOSFET with experimental data [30]. Fig 3(B) shows transfer characteristics comparison between HSB-BTFET and VPISDC-HSB-BTFET. As shown in Fig 3(B), the channel height of the HSB-BTFET is 5nm, and the height of the vertical channel of the VPISDC-HSB-BTFET is 1000nm. Comparing to the HSB-BTFET, the VPISDC-HSB-BTFET has lower current in the static state and the reverse biased state, which leads to lower static power consumption and lower reverse leakage current. Meanwhile, in the forward gate biased region, the VPISDC-HSB-BTFET generates a much higher ON-state current. The ON-state current of VPISDC-HSB-BTFET is increased from 2.5×10^−7^ A to approximately 2×10^−5^ A. The ON-state current increased about 80 times. More than that, VPISDC-HSB-BTFET achieves a lower subthreshold swing and a higher I_on_-I_off_ ratio compared to HSB-BTFET. Thereafter a more sensitive current driving ability is obtained by VPISDC-HSB-BTFET comparing to HSB-BTFET. Fig 3(C) shows the I_DS_-V_GS_ characteristic of VPISDC-HSB-BTFET with different vertical channel heights. The vertical channel height ranges from 50nm to 1μm. As the height of the vertical channel increases, the forward conduction current becomes larger at the same gate bias. because the vertical channel height increases, the contact area between the source-drain and the silicon increases, the total volume of the silicon region where the tunneling effect occurs increases, and the total tunneling current increases, resulting in the maximum forward conduction current at the interface between the source-drain and the silicon. Note that the reverse leakage current does not change evidently, due to that the source and drain contacts are not directly plug into the bottom of the silicon body, thereafter a certain distance is maintained between the main gate and the assistant gate, which effectively decreases the maximum electric field intensity in the silicon body for the reversed gate biased state, and prevent leakage current generated mainly by the band-to-band tunneling occurs in the region between main gate and assistant gate. As the vertical height increases, the effective channel length increases, more and more carriers pass through, and the current density increases, but not infinitely. When the height of vertical channel is above 500nm, the forward current does not increase significantly, thereafter there is an optimal value of the height of vertical channel, which is suggested to be about 1000nm.

**Fig 4:**
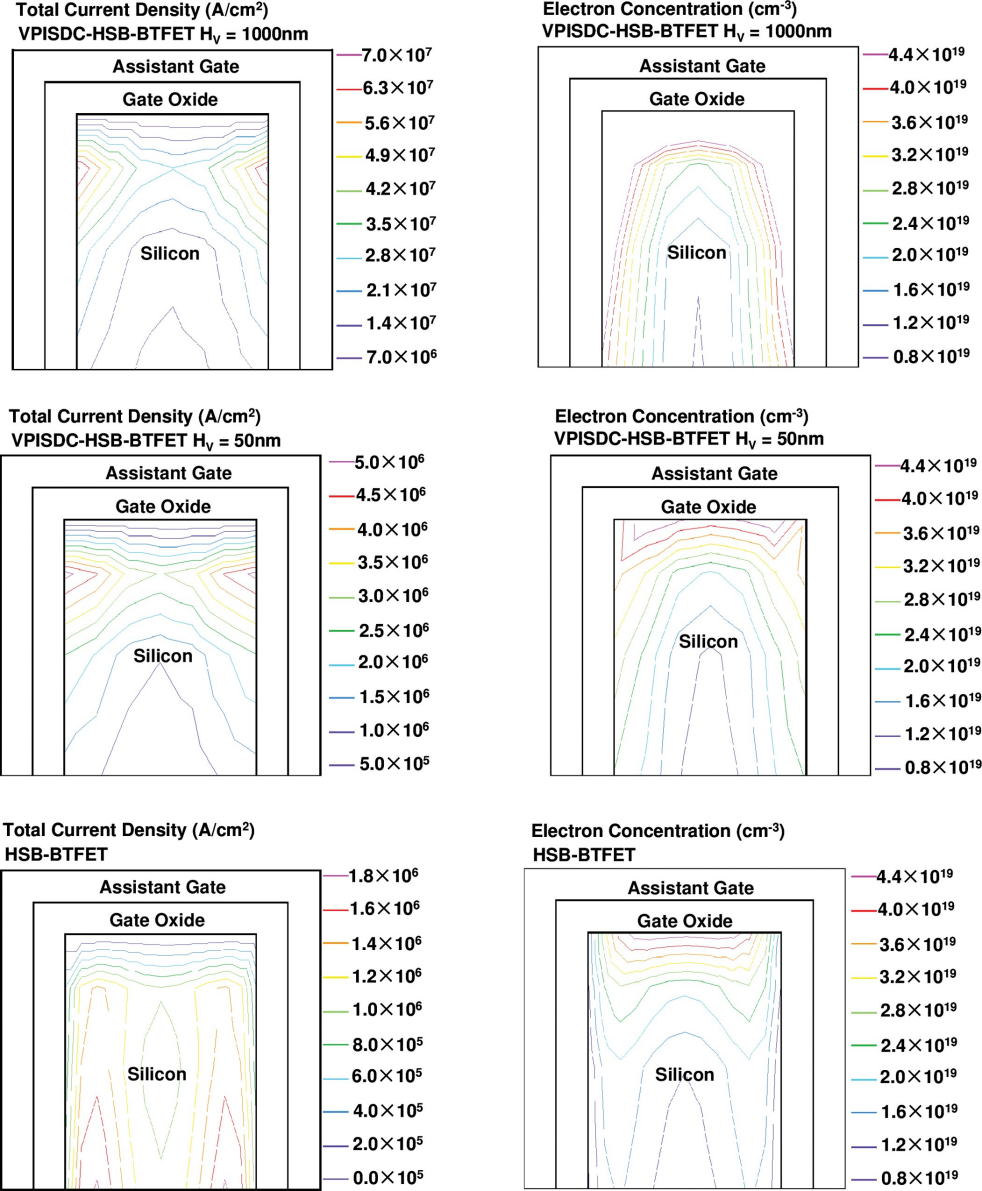
The total current density distribution and the electron concentration in the horizontal silicon body channel under the control of assistant gate in on state between (a) the proposed VPISDC-HSB-BRFET with 1000nm H_V_, (b) the proposed VPISDC-HSB-BRFET with 50nm H_V_ and (c) the previously proposed HSB-BRFET.

Fig 4(A)–4(C) show the comparison of total current density distribution and the electron concentration in the horizontal silicon body channel under the control of assistant gate in on state between the proposed VPISDC-HSB-BRFET with 1000nm H_V_, the proposed VPISDC-HSB-BRFET with 50nm H_V_ and the previously proposed HSB-BRFET, respectively. Under the same bias of assistant gate, the maximum value of current density of HSB-BTFET is nearly 2 orders of magnitude lower than the current density of VPISDC-HSB-BTFET with 1000nm H_V_. The peak electron concentration value of these three devices is almost the same (about 4.4×10^19^cm^-3^), however, due to the function of prolonged H_V_, the total area that reaches this peak electron concentration value is increased with the increasing of H_V_.

**Fig 5:**
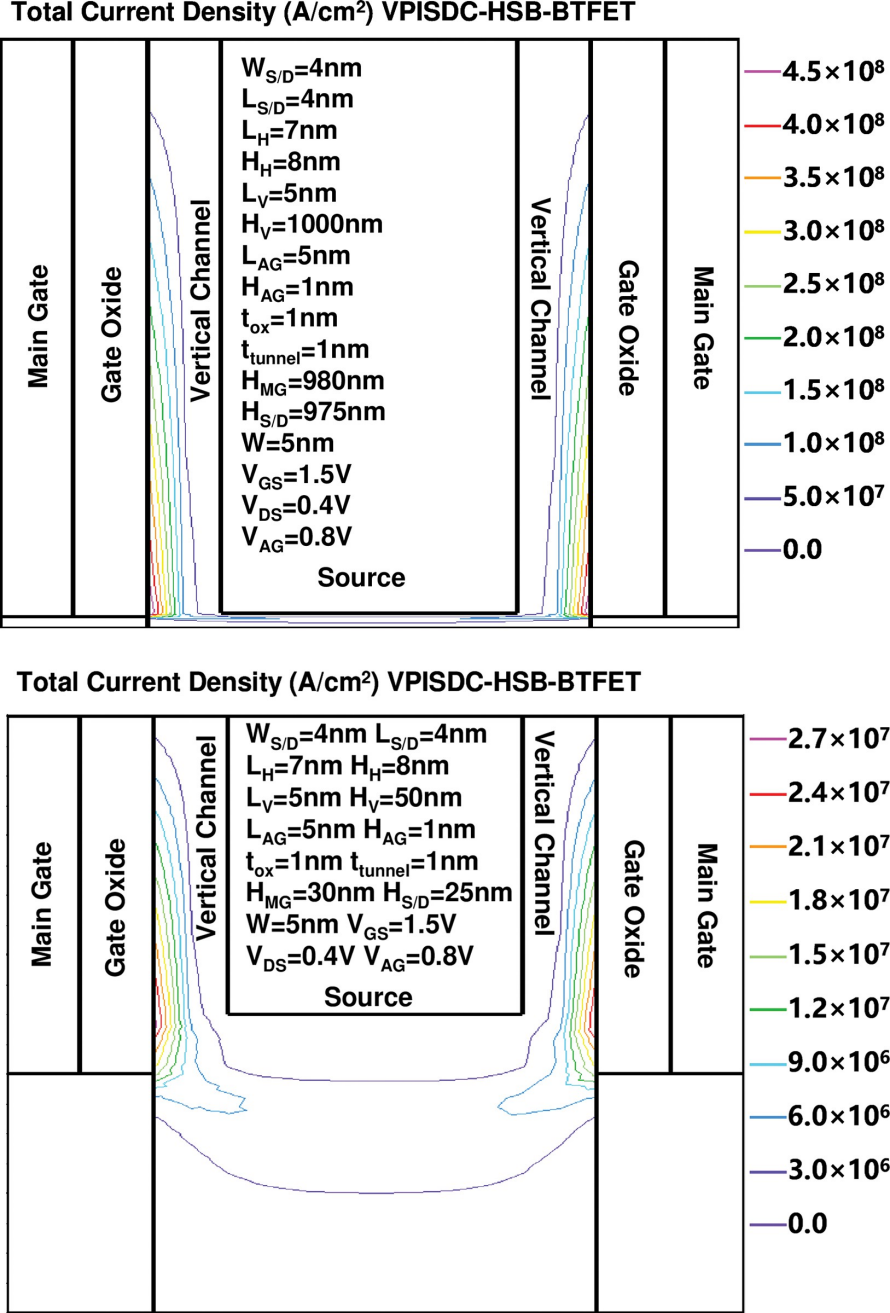
The total current density distribution in the vertical channel under the control of main gate in on state between (a) the proposed VPISDC-HSB-BRFET with 1000nm H_V_ and (b) the proposed VPISDC-HSB-BRFET with 50nm H_V_.

Fig 5(A) and 5(B) show the comparison of total current density distribution and the electron concentration in the vertical channel under the control of main gate in on state between the proposed VPISDC-HSB-BRFET with 1000nm H_V_, and the proposed VPISDC-HSB-BRFET with 50nm H_V_, respectively. As the height of the vertical channel increases, electrons generated by the tunneling effect from different heights converge to the bottom of the vertical channel. Therefore, as the height of the vertical channel increases, the sum of the generated tunnel charges and corresponding tunnel currents gradually increases. However, as the channel height increases, the distance required for tunnel electrons generated at higher vertical channel heights to flow from the source side to the drain side also increases, so the contribution of tunnel electrons to the total current will continue to decrease as the vertical channel height increases. Therefore, the recommended vertical channel height is about 1000 nm.

**Fig 6:**
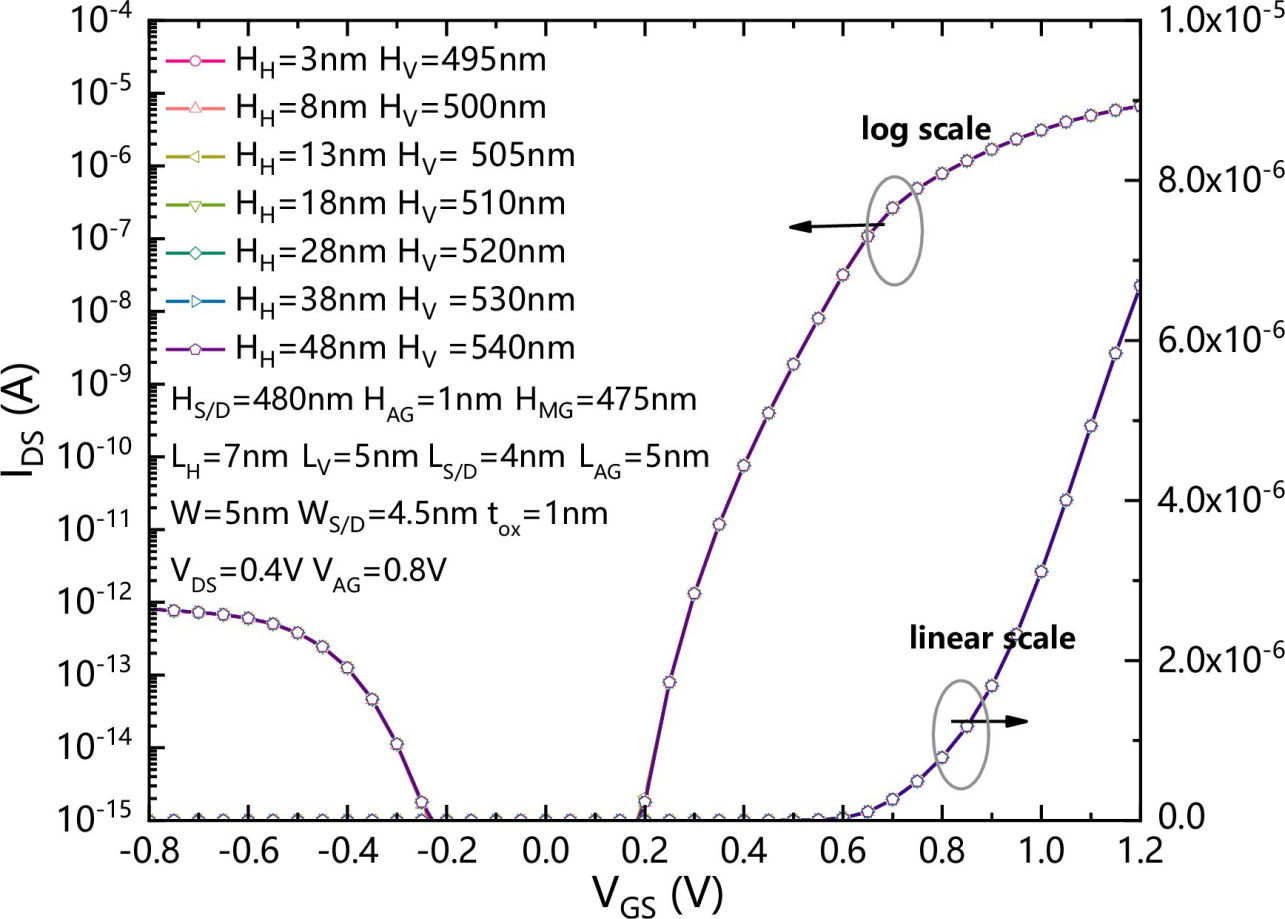
The comparison of transfer characteristics of the VPISDC-HSB-BTFET with different horizontal channel heights.

Fig 6 shows the comparison of transfer characteristics of the VPISDC-HSB-BTFET with different horizontal channel heights. Under the same V_AG_, the transfer characteristics are almost not affected by the changing of H_H_. This is mainly due to the sufficient reserved distance between MG and AG, and the potential difference between MG and AG is not sufficient to generate a strong enough electric field in the vicinity of AG to trigger a strong band to band tunneling effect.

**Fig 7:**
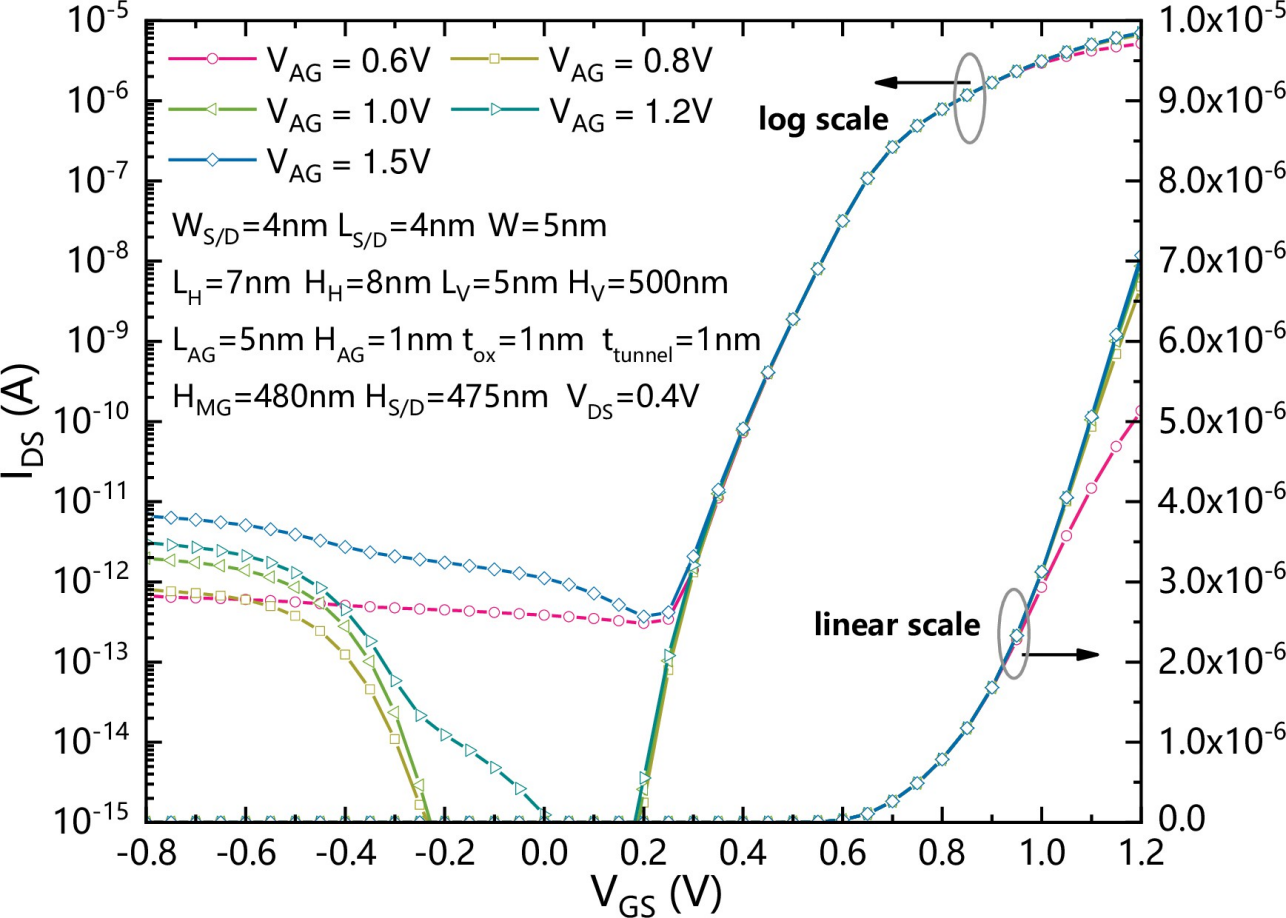
The comparison of transfer characteristics of the VPISDC-HSB-BTFET with different assistant gate voltages.

Fig 7 shows the comparison of transfer characteristics of the VPISDC-HSB-BTFET with different assistant gate voltages. The magnitude of the leakage current is strongly dependent on the voltage of the AG. A too small AG voltage can limit the carrier concentration in the horizontal channel and limit the formation of forward current, while a too large AG voltage can cause strong band bending in the silicon region controlled by the AG, resulting in excessive leakage current generation. With a gate oxide thickness of 1 nm, the recommended AG voltage is between 0.8 V and 1.2 V.

**Fig 8:**
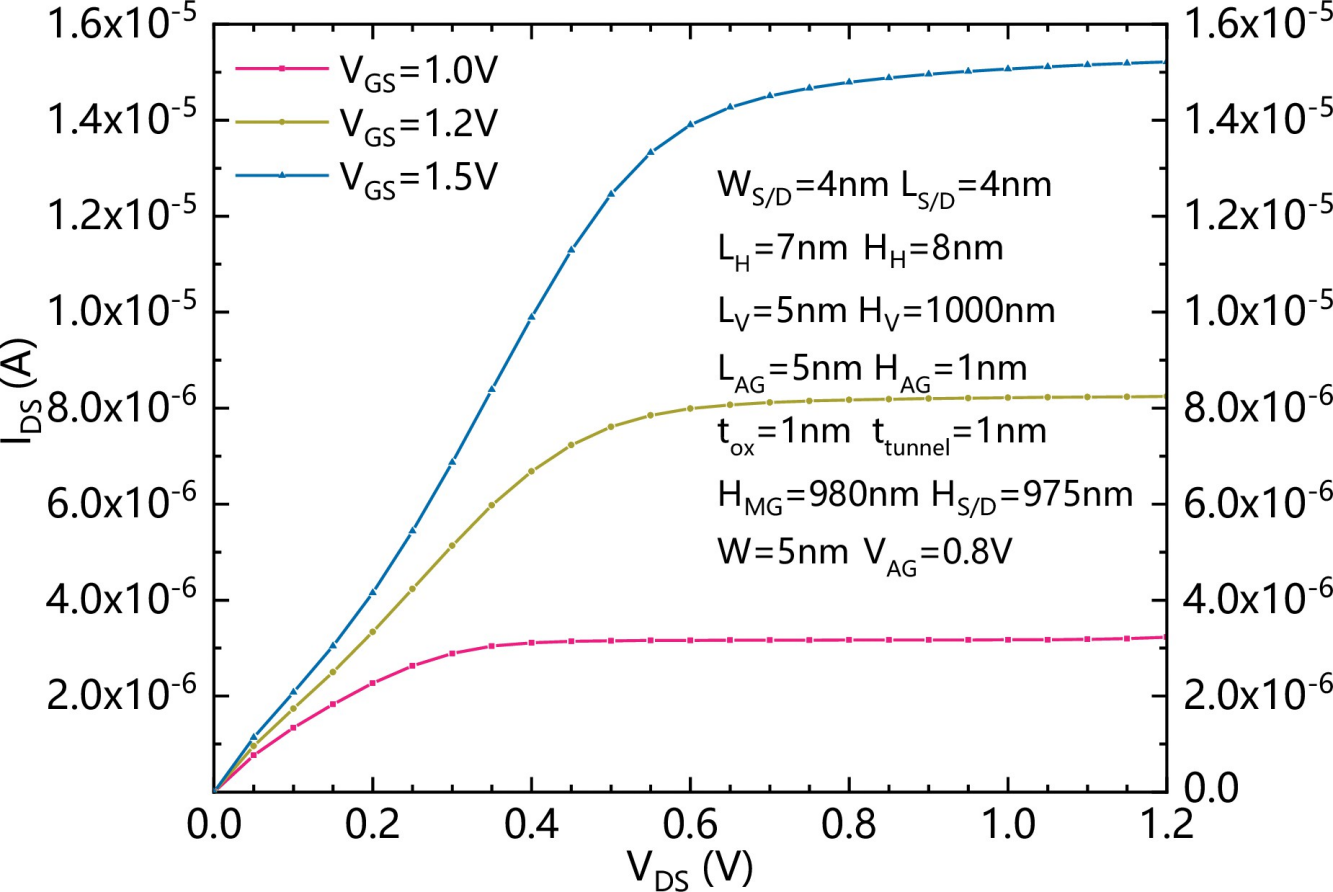
Output characteristics of the proposed VPISDC-HSB-BTFET with different V_GS_s.

Fig 8 shows the output characteristics of the proposed VPISDC-HSB-BTFET with different V_GS_s.

The forward saturation current reaches over 10^-5^A.

**Fig 9:**
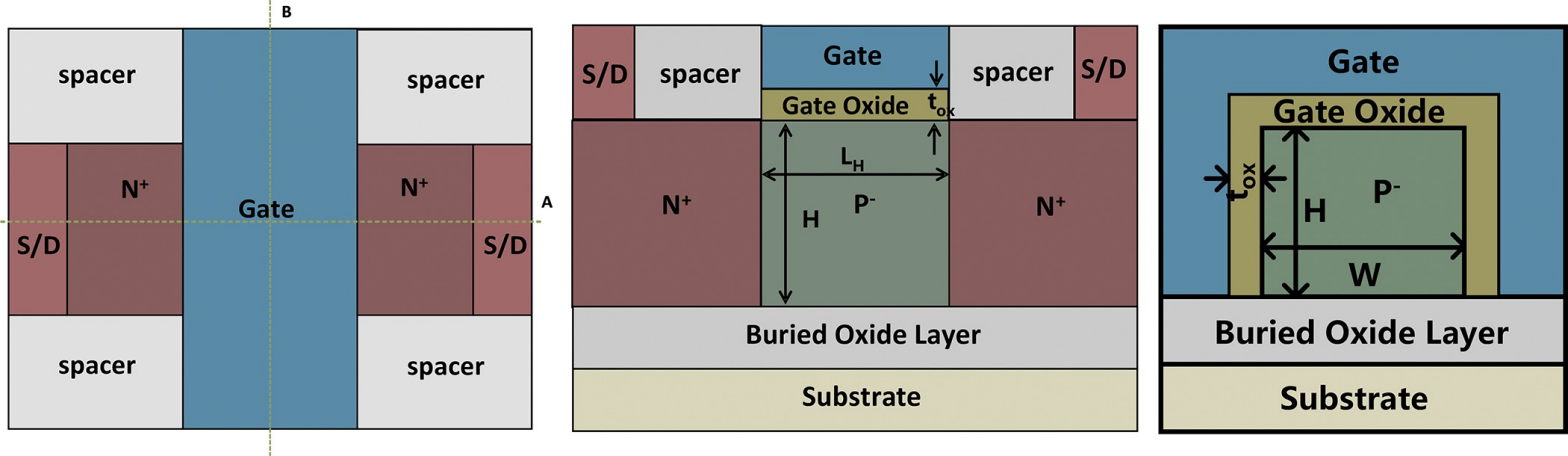
(a) Top view of the conventional FinFET, (b) cross views along cut line A in (a), (c) cross views along cut line B in (a).

Fig 9(A) shows a schematic view of the top view of a mainstream conventional fin field effect transistor (FinFET), Fig 9(B) and 9(C) are the cross view of conventional FinFET along the cut line A and B in Fig 8(A). In order to compare the performance of the two devices more reasonably, the structural parameters of the two devices are kept as consistent as possible. The same parameters such as horizontal channel length, width, and height (L_H_, W and H) and gate oxide thickness (t_ox_) are used.

**Fig 10:**
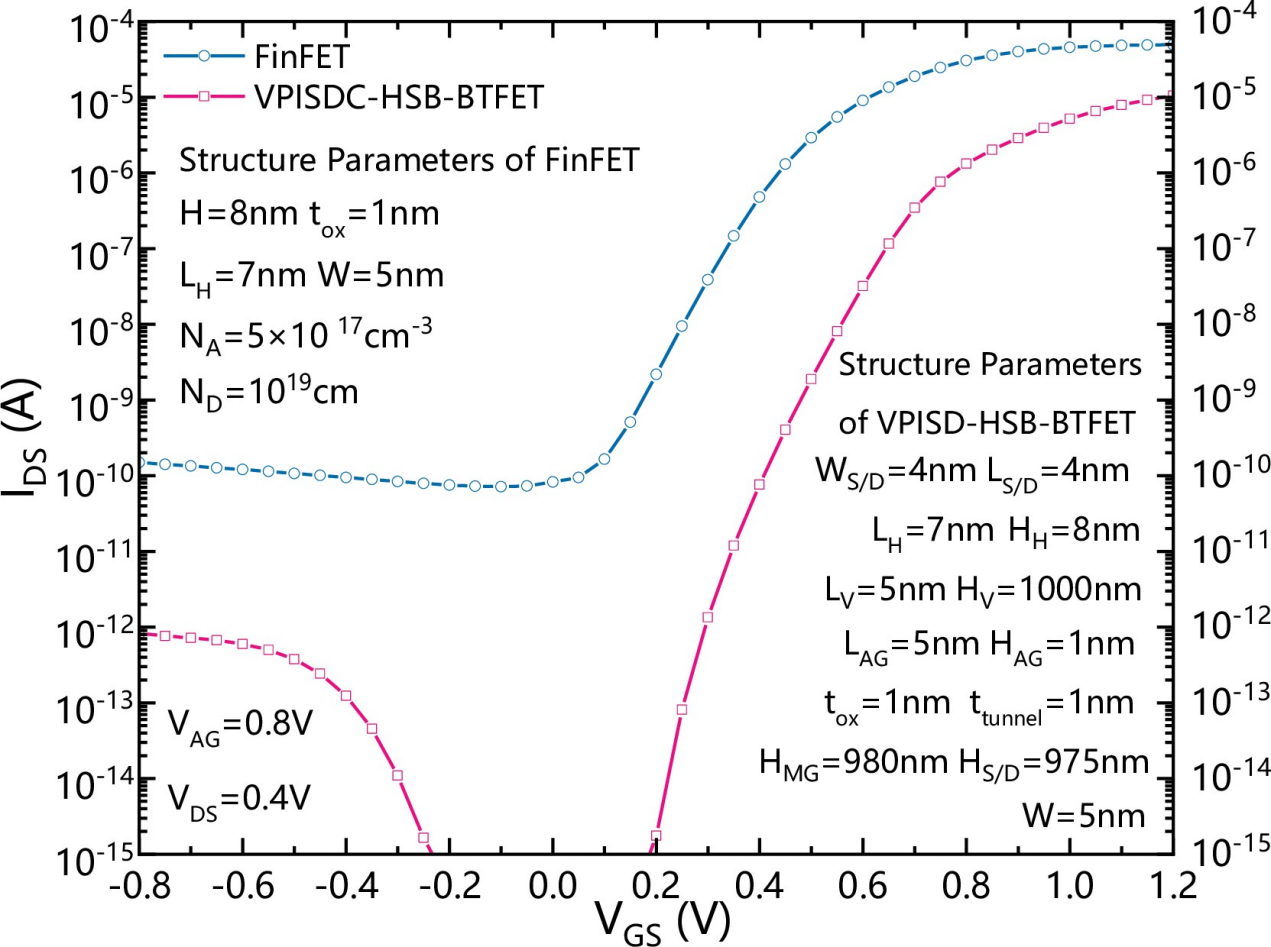
Transfer characteristics comparison between VPISDC-HSB-BTFET and FinFET.

The I_DS_-V_GS_ characteristics of conventional FinFET and VPISDC-HSB-BTFET are compared in the same simulation environment. Fig 10 shows the transfer characteristics comparison between VPISDC-HSB-BTFET and conventional FinFET. Both the channel height of the FinFET and the horizontal part the U-shaped channel VPISDC-HSB-BTFET are set to be 5nm, and the height of the vertical part of the VPISDC-HSB-BTFET silicon channel is 1000nm. Compared to conventional FinFET, the proposed VPISDC-HSB-BTFET has lower current in the lower forwardly biased region and reversely biased region, which leads to lower static power consumption and lower reverse leakage current. Meanwhile, compared to conventional FinFET, in the forwardly biased region, VPISDC-HSB-BTFET generates 10^-5^A ON-state current while FinFET generates 4**×**10^−5^. Therefore, the ON-state current and OFF-state current ratio (I_on_/I_off_ ratio) and the forward current and reverse current ratio (I_forward_/ I_reverse_ ratio) of the proposed VPISDC-HSB-BTFET are larger than those of FinFET. The average SS of the proposed VPISDC-HSB-BTFET is also reduced to less than 50 mV/dec, better than the ideal 63mV/dec SS of conventional FinFET.

Table 1 show the Comparison of I_on_/I_off_ ratio between highly sensitive devices and the mainstream FinFET technology. The advantage of the mainstream FinFET technology lies in the forward current due to the low source/ drain resistance. However, the increase in static leakage current due to size reduction cannot be ignored for nanoscale. The I_on_/I_off_ ratio of the proposed device is on an order of magnitude. The I_on_/I_off_ ratio of the proposed VPISDC-HSB-BTFET is in the order of magnitude of the I_on_/I_off_ ratio of the device proposed in Reference [19–21] and [23]. The I_on_ of the proposed VPISDC-HSB-BTFET is greatly increased, close to the value of mainstream FinFET.

**Table 1:** Comparison of I_on_/I_off_ ratio between highly sensitive devices and the mainstream FinFET technology.

Device	I_on_ (V_G_ = 1.2V)	I_off_ (V_G_ = 0V)	I_on_/I_off_ ratio
VPISDC-HSB-BTFET	~2×10^-5^A	~2×10^-16^A	~10^11^
HSB-BTFET	~2**×**10^-7^A	~2**×**10^-14^A	~2**×**10^7^
FinFET	~4**×**10^−5^ A	~10^−10^ A	~4**×**10^5^
Ref. [19]	~2**×**10^-6^A	~10^−17^ A	~2**×**10^11^
Ref. [20]	~2**×**10^-6^A	~10^−17^ A	~2**×**10^11^
Ref. [21]	~2**×**10^-6^A	~10^−17^ A	~2**×**10^11^
Ref. [22]	~8**×**10^-7^A	~10^-15^A	~8**×**10^8^
Ref. [23]	~2**×**10^-6^A	~10^−17^ A	~2**×**10^11^

**Fig 11:**
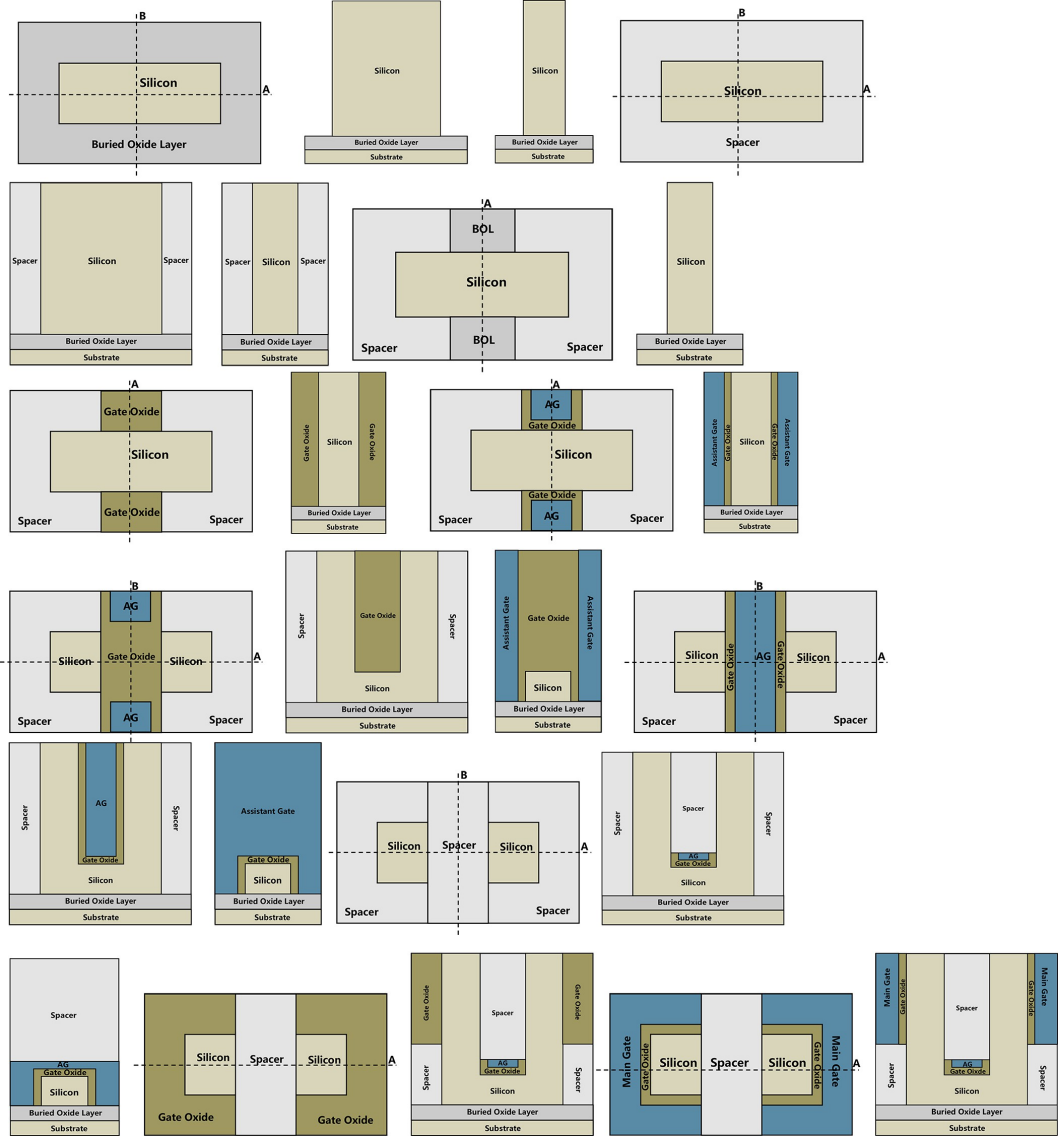


Fig 11 shows a brief manufacture process of the VPISDC-HSB-BTFET. As shown in Fig 11(A)–11(C), SOI wafers are prepared, and the monocrystalline silicon film above the SOI wafer is etched through photolithography and etching processes to remove the surrounding monocrystalline silicon film. As shown in Fig 11(D)–11(F), deposit insulating materials over the wafer, and then flatten the surface by a CMP process to initially form Spacer. As shown in Fig 11(G) and 11(H), remove the spacer located above and below the central portion of the silicon film through photolithography and etching processes to expose the BOL, leaving space for the gate insulating layer and assistant gate. As shown in Fig 11(I) and 11(J), remove the portion of the spacer both above and below the central portion of the silicon film, through photolithography and etching processes, then deposit insulating material with high permittivity such as HfO2 over the wafer, and then flatten the surface by a CMP process to initially form gate oxide. As shown in Fig 11(K) and 11(L), remove the inner portion of the gate oxide both above and below the central portion of the silicon film through photolithography and etching processes, then deposit metal or polysilicon over the wafer, and then flatten the surface by CMP process to initially form the assistant gate. As shown in Fig 11(M)–11(O), remove a certain thickness of the central portion of the silicon film through photolithography and etching processes, deposit insulating material with high permittivity such as HfO2 over the wafer, and then flatten the surface by a CMP process to further form gate oxide. As shown in Fig 11(P)–11(R), remove a certain thickness of the inner central portion of the gate oxide through photolithography and etching processes, deposit metal or polysilicon over the wafer, and then flatten the surface by a CMP process to further form gate oxide and assistant gate. As shown in Fig 11(S)–11(U), remove a certain thickness of the both the gate oxide and the assistant gate through photolithography and etching processes, deposit insulating material over the wafer, and then flatten the surface by a CMP process to further form the spacer. As shown in Fig 11(V)–11(X), remove a certain thickness of the spacer on both left side and right side of the silicon film through photolithography and etching processes, then deposit insulating material with high permittivity such as HfO2 over the wafer, and then flatten the surface by a CMP process to further form gate oxide. As shown in Fig 11(X) and 11(Y), remove a certain thickness of portions of the gate oxide on both left side and right side of the silicon film through photolithography and etching processes, then deposit metal or polysilicon over the wafer, and then flatten the surface by a CMP process to form the main gate. As shown in Fig 1(A)–1(C), remove a certain thickness of inner portions of the silicon film on both sides through photolithography and etching processes, then deposit metal such as Pt for n-type device or Er for p-type device over the wafer, after annealing, the source/drain electrode and the corresponding PtSi-Si/ErSi-Si high Schottky barrier interface is formed between the source/drain electrode and silicon film.

## Conclusion

In this paper, we propose a VPISDC-HSB-BTFET. Compared to HSB-BTFET, the proposed VPISDC-HSB-BTFET shows much better transfer characteristics such as more sensitive ON-state current driving ability and lower reverse leakage current. By comparing the transfer characteristics of VPISDC-HSB-BTFET under different vertical channel heights and analyzing the current density at the vertical channel heights of 50nm and 500nm, it can be concluded that as the vertical channel height increases, ON-state Current is obviously improved. Compared to conventional FinFET, the proposed VPISDC-HSB-BTFET shows lower static power consumption, higher I_on_-I_off_ ratio and lower SS. While avoiding expensive and complicated doping and annealing processes, the working performance of the device is maintained and improved. Compared to the mainstream FinFET technology, VPISDC-HSB-BTFET can realize more sensitive transfer characteristics, lower reverse bias gate-induced leakage current.

## Supporting information

S1 File (ZIP)

S2 File (ZIP)

S3 File (ZIP)

S4 File (XLS)

S5 File (XLS)

S6 File (XLS)

S7 File (XLS)
